# Defining High Value Elements for Reducing Cost and Utilization in Patient-Centered Medical Homes for the TOPMED Trial

**DOI:** 10.5334/egems.246

**Published:** 2019-05-03

**Authors:** Tracy Marie Anastas, Jesse Wagner, Rachel Lauren Ross, Bhavaya Sachdeva, LeAnn Michaels, Kimberley Gray, Katie Cartwright, David A. Dorr

**Affiliations:** 1 Oregon Health and Science University, US

**Keywords:** Primary Health Care, Patient-Centered Care, Utilization, Health Care Costs, Health Care Reform, Health Services Research

## Abstract

**Introduction:**

Like most patient-centered medical home (PCMH) models, Oregon’s program, the Patient-Centered Primary Care Home (PCPCH), aims to improve care while reducing costs; however, previous work shows that PCMH models do not uniformly achieve desired outcomes. Our objective was to describe a process for refining PCMH models to identify high value elements (HVEs) that reduce cost and utilization.

**Methods:**

We performed a targeted literature review of each PCPCH core attribute. Value-related concepts and their metrics were abstracted, and studies were assessed for relevance and strength of evidence. Focus groups were held with stakeholders and patients, and themes related to each attribute were identified; calculation of HVE attainment versus PCPCH criteria were completed on eight primary care clinics. Analyses consisted of descriptive statistics and criterion validity with stakeholder input.

**Results:**

2,126 abstracts were reviewed; 22 met inclusion criteria. From these articles and focus groups of stakeholders/experts (n = 49; 4 groups) and patients (n = 7; 1 group), 12 HVEs were identified that may reduce cost and utilization. At baseline, clinics achieved, on average, 31.3 percent HVE levels compared to an average of 87.9 percent of the 35 PCMH measures.

**Discussion:**

A subset of measures from the PCPCH model were identified as “high value” in reducing cost and utilization. HVE performance was significantly lower than standard measures, and may better calibrate clinic ability to reduce costs.

**Conclusion:**

Through literature review and stakeholder engagement, we created a novel set of high value elements for advanced primary care likely to be more related to cost and utilization than other models.

## Introduction

Americans pay more for health care than their international counterparts, yet the quality of care and health outcomes are mediocre in the United States [1]. Health care costs in this country are still rising, resulting in a call for approaches that will improve health care while curtailing costs. One of these efforts is a primary care-based model known nationally as the Patient-Centered Medical Home (PCMH) [2]. To obtain PCMH certification, primary care practices must demonstrate performance of particular services for their patients, such as improved access, care management, population management, and quality improvement. Under most PCMH implementations, practices can complete higher levels of elements from the models to receive higher incentives. To accommodate practice variations, PCMH models have many different elements as options; the most common PCMH model defined by the National Committee for Quality Assurance has more than 100 individual factors in various elements [3].

The results of PCMH trials are mixed, and intervention components have varied. Thus, more evidence is required to understand key approaches for success. A core criteria of success is whether they provide value, or more benefit for the additional costs to implement. A systematic review on PCMHs revealed that they have small positive impacts on patient experience, preventive care services, and staff experiences, but there were too few studies to estimate effects on clinical or economic outcomes [4]. Some studies have shown cost reductions, including the Geisinger, GroupHealth, and Intermountain Healthcare programs [5,6,7], but others have not consistently shown reductions, such as the Physician Group Practice program, the National Demonstration Project, and the Comprehensive Primary Care project [8,9,10]. As a result, we embarked to determine how PCMH definitions could be altered and to identify which elements were more likely to reduce cost and utilization to be considered “high value.”

For this study, Oregon’s medical home model, the Patient-Centered Primary Care Home (PCPCH), was chosen for evaluation and refinement because of the geographic location of the study, the cross-walk that existed between the model and other PCMH models, and the opportunity for uptake of the revised elements. In 2009, the Oregon Legislature established the program for primary care patients, intending to improve the Triple Aim—better population health, better experience of care, and reduced costs and utilization. Using the core elements of the standard—access, accountability, comprehensive, continuity, coordination, and patient and family-centeredness—we searched for papers that studied changes in primary care teams related to these elements that improved cost and utilization. In this paper, we describe how we refined PCPCH measures to identify “high value elements” (HVEs) for reducing cost and utilization with improved or equivalent patient health and experience of care. Second, we compare eight primary care clinics’ achievement on HVE and PCPCH measures at baseline for the Transforming Outcomes for Patients through Medical home Evaluation and reDesign (TOPMED) study. Identifying HVEs was an initial step in this pragmatic randomized controlled trial to define its intervention [11]. Clinics assigned to the intervention were instructed to select quality improvement goals related to HVEs, while control clinics selected goals related to PCPCH standards. We hypothesized that at baseline all clinics would pass fewer HVE measures than PCPCH measures because controlling costs and utilization is often more challenging than other practice transformation goals.

## Methods

### General Overview

The Oregon Health & Science University’s Institutional Review Board approved this study. To identify HVEs associated with cost and utilization reductions, we first performed an initial literature review on medical home model areas. Focus groups of stakeholders, experts, and patients identified elements from the literature review and assessed them for refinement and qualitative feedback. Once the HVE measures were identified, we evaluated eight primary care clinics on these measures and those of PCPCH for the TOPMED study. In this study, intervention clinics were encouraged to work on identified HVE measures, while control clinics worked on general PCPCH quality improvement. The full protocol for this study is published elsewhere [12]. After the trial was complete, we conducted a second literature review to identify additional evidence that suggested alternative versions of HVEs or enhanced their potential effectiveness.

### Initial literature review

We completed a literature review in MEDLINE from 1995–2013 searching for effect on cost and utilization in primary care from six broad core attributes—*Access to Care, Accountability, Comprehensive Whole Person Care, Continuity, Coordination and Integration, and Person and Family Centered Care*. The search strategy included each core attribute, primary care, cost or utilization, and included all study designs, from observational and cross-sectional to randomized controlled trials; we provide the terms and additional details in Appendix 1. From the initial abstracts, dual reviewers examined each article for relatedness to the review topic. Articles that were highly relevant from both viewers or through consensus were abstracted. From each article we abstracted the specific metrics tested, the relationship to PCPCH measures, and results on cost and utilization outcomes. The research team reviewed the abstractions and prepared a list of HVEs for the focus groups to provide feedback.

### Focus groups

A series of four focus groups were held to solicit feedback from patients, payers, clinic staff, and experts on the elements most likely to be related to cost and utilization. We recruited all stakeholders by posting information in clinics, on relevant listservs, and through networking. First, a group of adult patients with multiple chronic illnesses (n = 9) was recruited through local clinics, and a structured focus group was held with a trained facilitator. Each PCPCH standard and potential HVE were discussed. Patients were encouraged to discuss what might make a difference to them in terms of preventing exacerbations of their illnesses and avoiding emergency department (ED) visits and hospitalizations. The focus group was transcribed and reviewed by three researchers.

Themes from the patient focus group were then combined with the literature review findings. A multi-stakeholder panel of providers, payers, purchasers, researchers, and policy makers were recruited from local transformation work, from PCPCH listservs, and from known collaborators and convened (n = 12) to revise the HVE list based on their experiences, align HVEs with the PCPCH and other reform models, and discuss the feasibility of HVEs. HVEs included those that refined PCPCH standards as well as introduced new components not previously in the model. Furthermore, an insurer focus group (n = 22) discussed principles of policy reform related to HVEs, which allowed the research team to prioritize changes based on their ratings. Finally, staff (n = 6) from a clinic that participated in a 6-month TOPMED pilot period before the other clinics was interviewed. They provided feedback on the feasibility of HVEs and proposed measurements.

### Assessment

Eight clinics enrolled in the TOPMED study, and each clinic’s achievement of HVEs was measured. The study included the usage of a health information technology (HIT) tool, the Integrated Care Coordination System (ICCIS), which was designed for care management tracking. A PCPCH Attestation Tool module was added with the standard PCPCH measures and the HVEs. Measurement consisted of two phases. First, electronic health record (EHR) data was drawn into the tool where possible and summarized for each quantitative element. Second, a practice facilitator assisted clinic staff in a 1–2 hour in-person meeting to explain and assess additional elements, and calculate a baseline measure. Clinics also reviewed the quantitative measurements and were allowed to provide more specific evidence to adjust these calculations.

### Second literature review

After completing the TOPMED trial, a second literature review was performed to update the original search with new or missed articles for future refinement of measures. A broad search was performed on the six PCPCH core attributes, and individual HVEs were also specifically searched. Articles were once more abstracted for metrics, quality, and outcomes. Abstraction from all articles is included in Appendix 2.

## Results

The literature review identified 2,126 abstracts, of which 22 articles met criteria and were abstracted. Excluded articles were about primary care redesign without a focus on specific elements, were thought or opinion papers, or involved different settings.

Table 1 highlights the articles abstracted with their related PCPCH core attributes. 14 of the 22 articles we found had significant positive outcomes of cost and utilization (p < .05) [13,14,15,16,17,18,19,20,21,22,23,24,25,26]; four had a non-significant trend towards positive outcomes [27,28,29,30]; and four had no change [31,32,33,34]. The studies for each core attribute are highlighted below, along with input from focus groups, the specific HVE measurements, and the baseline performance of the clinics. Descriptions of HVE measures can be found in Table 2.

**Table 1:** Summary of included studies and effect on outcomes.

Core Attributes	Study design	Count of studies	Outcomes: Cost & Utilization			Related Articles*	High Value Element Number & Examples

			✓	+	~	1	1: Reminders about comprehensive services due

**Comprehensive**	RCT	2	2	0	0		

			✓	+	~	3	3: After hours access, 3rd next available appointment, Tracking responses to requests

**Access**	Observational	1	1	0	0		
	Cross-sectional	1	1	0	0		

			✓	+	~	4	4: Care Plan Utilization, Advance Directive Utilization

**Coordination & Integration**	RCT	3	1	1	1		
	Observational	1	0	1	0		

			✓	+	~	2	3: Clinical information exchange, Utilization follow-up and prevention

**Continuity**	RCT	5	2	0	3		
	Quasi-experimental	1	0	1	0		
	Observational	2	2	0	0		
	Cross-sectional	1	1	0	0		

			✓	+	~	6	1: Education & Self-Management Support

**Person & Family Centered Care**	RCT	2	1	1	0		
	Quasi-experimental	2	2	0	0		
	Observational	1	1	0	0		

	**Total**	**22**	**14**	**4**	**4**	**14**	

RCT: Randomized Controlled Trial; ✓: statistically significant positive outcomes; +: Trending positive outcomes, not statistically significant; ~: no effect; no studies we identified demonstrated a negative impact on outcomes. * The count of “Related Articles” were not found in the initial review. They were found in the second review that was conducted based on stakeholder feedback. 7 of the 14 are systematic reviews representing 231 studies. 1 of the 7 is a review of reviews representing 17 systematic reviews including 390 studies.

**Table 2:** HVE measure descriptions.

PCPCH Core Attributes	HVE Measure	Level 1	Level 2	Level 3

**Access**	After Hours Access	Offers access to in-person care at least 12 hours weekly outside traditional business hours.		
	Tracking 3rd Next Available Appointments	Tracks 3rd next available appointments.	Meets a benchmark on 3rd next available appointments.	
	Tracking/responding to electronic requests	Able to receive and respond to electronic requests.	Able to track electronic request response times.	Provides a response to online or electronic queries within two business days.

**Comprehensive Whole Person Care**	Reminders	Uses patient information, clinical data, and evidence-based guidelines to generate lists of patients who need reminders and to proactively remind patients/families/caregivers and clinicians of needed services.	Tracks the number of eligible patients who were sent appropriate reminders.	Sends appropriate reminders to at least 20% of all eligible patients.

**Continuity**	Clinical Information Exchange	Exchanges structured clinical information and tracks critical elements (e.g., hospitalizations).		
	Utilization Follow-up	Follows up on patient hospitalizations and ED visits 30% of the time (when they have the information).	Follows up on patient hospitalizations or ED visits 70% of the time (when they have the information).	Follows up on patient hospitalizations and ED visits 70% of the time (when they have the information).
	Utilization Prevention	Selects and reviews utilization measures and goals most relevant to their overall patient panel, or an at-risk patient population.	Shows improvement or meets a benchmark in utilization metrics on measures closely linked to utilization.	

**Coordination & Integration**	Care Plan Utilization	Reports data on care plans provided to high-risk patients.	Provides care plans to >25% of high-risk patients.	Provides care plans to >50% of high-risk patients.
	Advance Directive Utilization	Tracks offers of advance directives to patients over 65.	Offers advance directives to at least 30% of patients over 65.	Offers advance directives to at least 50% of patients over 65.
	Performance Data Utilization	Uses performance data to identify opportunities for improvement and acts to improve clinical quality, efficiency and patient experience.		
	Care Coordination Outreach	Care coordination outreach reaches 25% of high-risk patients.	Care coordination outreach reaches 50% of high-risk patients.	

**Patient & Family Centered**	Education and Self-management Resources	More than 10% of all unique patients are provided patient-specific education resources.	More than 10% of all unique patients are provided patient-specific education resources and self-management services.	

*Comprehensive care* defines a foundational aspect of primary care in that the breadth of services offered meet most initial acute, chronic, and preventive needs. This organizational characteristic was broad and did not yield many specific articles. In interviews, patients described comprehensiveness as “more dimensional than just a single primary care doctor,” and “[a] team approach [that] seems like a good use of resources.” Hence, general team-based enhancements and an emphasis on meeting chronic and preventive needs were selected for focused searches. For team-based care, two studies were identified, with both finding significant reductions in cost. One study described a team-based approach using social workers and registered nurses to augment PCP efforts for chronically ill seniors; the intervention team kept costs stable while control patients’ costs increased [13]. Unutzer et al. demonstrated in the IMPACT program that nurse care managers and consult psychiatrists engaged with primary care can lower utilization and costs for older adults with depression [14].

Reminders about preventive and chronic illness care were highlighted by patients and experts to keep at-risk patients close. Subsequently, an HVE goal for sending reminders to at least 20 percent of patients due for chronic and preventive services was developed. A post-trial targeted search revealed a systematic review on reminders that found 42 out of 51 studies had positive outcomes across preventive and chronic illnesses [35].

*Access* refers to enhancing patients’ ability to receive care, often by expanding available appointment schedules and/or reducing providers’ response time when communicating with patients. Two articles were found, showing significant cost and utilization decreases. Focus groups highlighted the importance of access as patients explained, “My experience… [is that I] may or may not receive a returned phone call,” and “A lot of patients complain about scheduling and the availability of doctors.”

The two studies focused on expanded, or “after hours,” access. O’Malley found an absolute difference of –7.3 patients per hundred with one or more ED visits in clinics with expanded access [16], while Lowe reported .80 relative rate of ED visits for clinics holding 12 or more office hours outside of normal business hours [15]. One systematic review focusing broadly on advanced access identified eight studies measuring third next available appointment [36]. These eight studies showed a mixed effect on utilization.

The first HVE selected matched Lowe’s definition: “Offers access to in-person care at least 12 hours weekly outside traditional business hours” [15]. Based on patient suggestions, a second HVE was created to target improved appointment availability. Despite a mixed utilization effect, third next available was adopted as an HVE metric based on stakeholder input. Finally, patients expressed that the response time to electronic requests was a crucial element in preventing ED utilization. Therefore, the standard measure and benchmark from the Meaningful Use EHR certification program was used as the third access HVE: 48-hour response time after an electronic request.

*Coordination and integration* of care allows fragmented health care settings and teams to communicate about intended care plans, make adjustments, and bring care elements together either virtually or practically. Four studies were found related to coordination and integration.

Two studies focused on the use of nurse care managers. Boult et al. studied small panels of high-risk patients, and showed trends of decreased admissions and readmissions [27], while with a larger set of patients with complex conditions including diabetes, Dorr et al. significantly decreased utilization with absolute hospitalization reductions of 4 percent and 8 percent at one and two years [17]. Contrarily, Toseland et al. [31] studied outpatient geriatric evaluation and management for frail elders but found no change in cost or utilization over eight months. Coupled with other team-based changes, a focus on risk stratification, care planning, and goal setting was identified from these articles and experts. A secondary search of non-abstracted articles found related elements in non-primary care settings. Newton et al. created care plans for 32 patients frequently seen in the ED and found significant reductions in ED visits and hospital admissions [37]. Another cohort study on frequent users of the ED examined the effect of care plans and demonstrated a significant reduction in ED visits [38]. Stakeholders reinforced the need to reach a substantial population of those at-risk to see changes. Consequently, we created two HVEs based on identifying high-risk patients and providing goal setting, care planning, and care coordination outreach for 25–50 percent of the at-risk population.

For advance care planning, Nicholas et al. showed advance directive use in high spending regions was associated with lower costs [28]. Several other related papers did not meet full inclusion criteria; nevertheless, their findings provide evidence supporting advance care planning. For instance, one demonstrated that patient satisfaction increased but did not measure utilization with advance directives [39], while another found that the Oregon Physician Orders for Life-Sustaining Treatment (POLST) registry led to 44 percent of POLST orders altering the treatment provided to patients based on their wishes [40]. Use of advance directives and advance care planning was also reinforced by experts and providers, and for the advance directive HVE, 30 percent and 50 percent benchmarks were added to the extant PCPCH measure of “Offering and recording advance directives for patients 65 and older.”

*Continuity of care* refers to health care teams partnering with patients and families to provide consistent care over time. Nine articles were found; six demonstrated a decrease in utilization [18,19,20,21,22,29], and three showed no difference [32,33,34]. Wasson et al. showed patients randomly assigned to see the same primary care provider (‘continuity’) in follow-up versus a random provider had significantly less utilization, especially for emergent issues (20 percent continuous versus 39 percent for discontinuous), and higher satisfaction [18]. Ionescu-Ittu and colleagues analyzed data from 95,173 patients finding that low levels of continuity and little to no use of a primary care provider (PCP) was related to increased ED visits (RR 1.24–1.46) [22]. Furthermore, readmission reduction programs from the hospital or ED to home have strong evidence of effectiveness [41]; however, the role of primary care follow-up is unclear [42]. Two studies showed primary care teams could reduce repeat, unplanned ED visits through structured follow-up by phone or visit [19,20]. Frisse et al. found providing health information data to the ED from outside sources reduced predicted hospital admissions (OR 0.48 for HIE used versus not used for admission, p < 0.001) [21]. However, Overhage et al. did not find improved outcomes when the EHR from ambulatory providers was shared with ED physicians [32], and neither Gurwitz et al. [33] nor Balaban et al. [34] showed structured information at hospital discharge reduced readmissions significantly.

Despite the mixed results, stakeholders emphasized the importance of exchanging information, noting that “It would be helpful if clinics could advance their ability to receive this information electronically and develop a tracking system … [to] coordinate care of patients across venues.” Three HVEs were created for continuity, with one chosen on the criterion of “Exchanges structured clinical information and tracks critical elements (e.g., hospitalizations).” Similarly, patients noted that if they “go to another hospital, their PCP is unaware of their visit.” Thus, monitoring unplanned utilizations was suggested as a second measure. Finally, follow-up after unplanned utilization for a substantial proportion of the population (30 percent and 70 percent) was selected as a stepped HVE measure based on the literature.

*Person- and family-centered care*, focusing on the needs, preferences, values, and goals of patients and their families and caregivers, is a tenet of primary care practice [43], and one HVE was refined from it. Five studies were identified; four had significant impacts on cost and utilization [23,24,25,26], while one had a trend towards positive improvement [30]. Bertakis and Azari recorded 509 patient consultations with 105 providers and demonstrated higher patient-centered communication led to fewer hospitalizations (0.11 vs. 0.25 per patient-year in high vs. lower patient-centered communication) and a decrease in total costs [24]. DeWalt et al. found patients with congestive heart failure given self-management education had non-significant lower combined outcomes of hospitalizations and death versus control (42 percent versus 61 percent) [30], and Coleman et al. found older adults with chronic illness given self-management intensive group visits had fewer ED visits than usual primary care (0.61 versus 1.08 per person during the two-year study period) [23]. Finally, Greisinger et al. found structuring diabetes care to provide consistent education and monitoring reduced hospitalizations and costs [26]. All stakeholders noted this element as important; patients said, “It’s important that the patient’s input is important to the doctor… in considering my goals.” Additionally, studies of health empowerment [44], patient activation [45], shared decision making [46], self-management support [47], motivational interviewing [48], and goal setting [49] provided helpful metrics and demonstrated improvements in health. From a synthesis of these studies and stakeholder input, providing self-management support and educational resources to 10 percent of high-risk patients was identified as an HVE.

### PCPCH and HVE clinic assessment

After the HVEs were defined, measurement of both the standard elements of the PCPCH and the HVEs were completed in eight TOPMED-enrolled clinics. Table 3 demonstrates the average passing percentages (meeting the defined expectations) for the PCPCH standards, showing good discrimination for HVEs versus standard PCPCH. The full criterion for HVEs and their measurement specifications and relationship to PCPCH standards are available in Appendix 3. On average, clinics were passing 87.9 percent of PCPCH standards. For the new HVEs, only 38 percent of elements were passed. Higher levels of elements represent harder to achieve measures or benchmarks; for PCPCH, 60.5 percent were passing the highest level three, while only 19 percent were passing the same level of HVEs.

**Table 3:** Percent passing initial PCPCH and HVE defined models.

PCPCH Core Attributes	HVE/PCPCH	Measures*	# of Levels	% of clinics passing Level 1	% of clinics passing Level 2	% of clinics passing Level 3

**Comprehensive Whole Person Care**	**PCPCH**	Preventive Services	1	75%		
		Mental Health, Substance Abuse, and Developmental Services*	2	N/A	100.00%	87.50%
		Comprehensive Health Assessment & Intervention	1	100%		
	**HVE**	Reminders	3	75%	12.50%	12.50%

**Access**	**PCPCH**	In-Person Access	3	100%	62.50%	12.50%
		After Hours Access—4 hours	1	75%		
	**HVE**	After Hours Access—12 hours	1	37.50%		
		Tracking 3rd Next Available Appointments	2	37.50%	0%	
		Tracking/responding to electronic requests	3	62.50%	50%	50%

**Accountability^§^**	**PCPCH**	Performance & Clinical Quality Improvement*	2	N/A^†^	75%	37.50%

**Continuity**	**PCPCH**	Personal Clinician Assigned	1	N/A	N/A	100%
		Personal Clinician Continuity	1	N/A	N/A	62.50%
		Clinical Information Exchange—shares electronically	1	N/A	N/A	100%
	**HVE**	Clinical Information Exchange—shares & tracks electronically	1	37.50%		
		Utilization Follow-up	3	37.50%	37.50%	12.50%
		Utilization Prevention	2	0%	0%	

**Coordination & Integration**	**PCPCH**	Population Data Management	2	100%	100%	
		Electronic Health Record	1	N/A	N/A	87.50%
		Care Coordination—describes process	2	100%	100%	
		Test & Results Tracking	1	62.50%		
		Comprehensive Care Planning—demonstrates ability	1	N/A	87.50%	
		Referral & Specialty Care Coordination	3	100%	100%	100%
	**HVE**	Care Plan Utilization—for a % of high-risk patients	3	50%	37.50%	12.50%
		Advance Directive Utilization	3	62.50%	12.50%	12.50%
		Performance Data Utilization	1	75%		
		Care Coordination Outreach—for a % of high-risk patients	2	50%	37.50%	

**Patient & Family Centered**	**PCPCH**	Education & Self-Management Support—documents	1	100%		
		Experience of Care	3	100%	62.50%	0%
	**HVE**	Education & Self-management Resources—for % of patients	2	0%	0%	

	**Totals**	**% likelihood a clinic is passing**		**91%**	**85.90%**	**60.90%**
		**% likelihood a clinic is passing**		**44%**	**19%**	**20%**

* These measures contain a Must Pass level. There are eight additional Must Pass measures without additional level. † Each level for PCPCH is 5 points, 10 points, and 15 points for levels 1, 2, 3 and respectively. In some cases, levels are skipped and the first levels are worth 10 points for 15 points, hence the N/A. § There were no HVEs associated with Accountability.

## Discussion

Engagement in primary care alone provides some benefit to patients in reducing cost and utilization; however, redesigned primary care models like the PCMH or the PCPCH do not consistently show an increased benefit. We were successful in better understanding and working to close this gap by searching the literature and engaging a set of stakeholders and patients to identify a set of 12 HVEs related to these models, which are more likely to reduce cost and utilization and improve the Triple Aim. From utilization management and follow-up to intensive care coordination and care management of at-risk patients, these elements were not consistently performed in the group of clinics despite their high performance in the standard PCPCH model. This gap indicates that this evidence- and expert-based approach has the potential to refine current models and increase the likelihood that implementation will reduce cost and utilization.

The opportunity of health reform relies on more effective and efficient care enabling people to avoid adverse health outcomes that lead to expensive, unnecessary care. As the U.S. spends the largest percentage of its GDP on health care compared to similar nations [50], the opportunity to reduce wasteful spending is great. Given our fragmented system; however, it has been hard to find models that consistently work. By measuring more precisely the HVEs that may reduce this utilization, we allow many disparate programs and systems to set standard benchmarks for improvement while still allowing local innovation. This is especially true for clinics outside of large delivery systems, which lack the advantage of centralized planning, and the ability to shift external resources to meet new reforms. These HVEs can all be measured at the clinic level, and, with stakeholder input, were considered feasible to measure and achieve.

*Limitations of the study*. The approach to identify articles across each PCMH domain led to difficulty finding articles directly addressing primary care, the topic area, and the outcomes of cost and utilization. For instance, many other studies related to access (e.g., nurse consultation lines [51,52], patient portals [53,54,55]) were available but did not meet inclusion criteria due to the larger context of these studies (e.g., in health systems, rather than solely in primary care practices). This led to targeted searches suggested by stakeholders or from references and related sources, which did not allow us to perform a systematic review in every area. We accounted for this by repeating the search post-study and thoroughly cross-checking articles for salient references and relatedness. Moreover, the PCPCH definition changed after the study began, with three of 12 HVEs added as PCPCH standards, so the initial comparison is less immediately valid. Similarly, other health reform initiatives, especially the Comprehensive Primary Care initiative (and its Plus successor) have very similar elements, including providing high-risk patients with care plans and care coordination elements. We find this national overlap confirming, especially in light of our Oregon PCPCH focus.

Future research may work to design and implement these elements more formally for value-based payment reform, and use them as indicators for best likelihood to affect cost and utilization from the primary care setting. The goal of the federal government to move 90 percent of all payments into value-based programs by 2018 [56] is encouraging in that we will see a larger move away from volume-based reimbursement and towards rewarding HVEs of care.

## Conclusion

Through a combination of literature review and stakeholder engagement, we created a related but novel set of high value elements for advanced primary care likely to be more related to cost and utilization than other models. Initial evaluation of this set of conditions showed lower percentages passing than one medical home model in Oregon, indicating significant opportunity for testing.

## Additional Files

The additional files for this article can be found as follows:

10.5334/egems.246.s1 Appendix 1. Search strategy and results.

10.5334/egems.246.s2 Appendix 2. Literature review abstracted results.

10.5334/egems.246.s3 Appendix 3. HVE and PCPCH standards descriptions.
