# Supplementary material for: Defining High Value Elements for Reducing Cost and Utilization in Patient-Centered Medical Homes for the TOPMED Trial
Source: EGEMS (Wash DC). 2019 May 3;7(1):20. doi: 10.5334/egems.246 (PMC6498873; doi:10.5334/egems.246)
Supplement: Appendix 1. — Search strategy and results. [file egems-7-1-246-s1.pdf]

## Appendix 1. Search strategy and results

For the literature review and update, we created and iteratively improved a series of search strategies. These are provided in the table below. First, we used standard terms – filtering on clinical trial, primary health care, and on MeSH terms related to cost and utilization, as shown in Table A1. Then, we added terms related to each of the subsets, attempting to use the most sensitive terms and adding refinements to make the terms more specific. Table 1 contains these iterative queries. We also used the find related articles in MEDLINE to expand our searches.

Table A1. Iterations of search terms

| **Standard** | **Search-clinical trial filter for all** |
|---|---|
| Access | MeSH and keyword- "primary health care" AND "cost and cost analysis" OR "utilization review" AND "health services accessibility" |
| | MeSH- "primary health care" AND "cost and cost analysis" OR "utilization review" AND "health services accessibility" without clinical trial filter |
| Accountability | MeSH exploded overall and focus on "quality improvement" with subheadings "Organization" and "trends" |
| Comprehensive | Exp "Cost and Cost Analysis" + exp "utilization review"+ "primary health care" (economics, methods, organization and administration, stats) |
| Continuity | All MeSH- "primary health care" AND "cost and cost analysis" OR "utilization review" AND "continuity of patient care" |
| | MeSH- focus "primary health care" & explode "continuity of patient care" |
| Coordination/Integration | MeSH and keywords- "primary health care" AND "cost and cost analysis" OR "utilization review" AND "coordination" (non-MESH) |
| | MeSH and keywords- "primary health care" AND "cost and cost analysis" OR "utilization review" AND "integration" (non-MeSH) |
| | All MeSH- "primary health care" AND "cost and cost analysis" OR "utilization review" AND "delivery of health care, integrated" |
| Person & Family Centered Care | MeSH and keywords- "primary health care" AND "cost and cost analysis" OR "utilization review" AND "patient-centered care" |
| | MeSH and keywords- "primary health care" AND "cost and cost analysis" OR "utilization review" AND "care giver" |
