# Supplementary material for: Defining High Value Elements for Reducing Cost and Utilization in Patient-Centered Medical Homes for the TOPMED Trial
Source: EGEMS (Wash DC). 2019 May 3;7(1):20. doi: 10.5334/egems.246 (PMC6498873; doi:10.5334/egems.246)
Supplement: Appendix 2. — Literature review abstracted results. [file egems-7-1-246-s2.pdf]

Appendix 2: HVE and PCPCH standards descriptions

PCPCH Core Attributes	HVE/PCPCH	Measure	Level 1	Level 2	Level 3
**Access**	**PCPCH**	In-Person Access	Surveys a sample of its population on satisfaction with in-person access to care and reports results.	Surveys a sample of its population on in-person access to care using one of the Consumer Assessment of Healthcare Providers and Systems (CAHPS) survey tools	Surveys a sample of its population using one of the CAHPS survey tools, and meets a benchmark with patient satisfaction in access to care.
		After Hours Access—4 hours	Offers access to in-person care at least 4 hours weekly outside traditional business hours.		
	**HVE**	After Hours Access—12 hours	Offers access to in-person care at least 12 hours weekly outside traditional business hours.		
		Tracking 3rd Next Available Appointments	Tracks 3rd next available appointments.	Meets a benchmark on 3rd next available appointments.	
		Tracking/responding to electronic requests	Able to receive and respond to electronic requests.	Able to track electronic request response times.	Provides a response to online or electronic queries within two business days.
**Accountability**[§]	**PCPCH**	Performance & Clinical Quality Improvement*	N/A	Tracks and reports to the OHA two measures from core set and one measure from the menu set of PCPCH Quality Measures.	Tracks, reports to the OHA and meets benchmarks on two measures from core set and one measure from the menu set of PCPCH Quality Measures.
**Comprehensive Whole Person Care**	**PCPCH**	Preventive Services	Offers or coordinates 90% of recommended preventive services (Grade A or B USPSTF Recommended Services and/or Bright Futures periodicity guideline).		

**Comprehensive Whole Person Care**	**PCPCH**	Mental Health, Substance Abuse, and Developmental Services	N/A	Documents direct collaboration or co-management of patients with specialty mental health, substance abuse, or developmental providers.	Documents actual or virtual co-location with specialty mental health, substance abuse, or developmental providers.
		Comprehensive Health Assessment & Intervention	Documents comprehensive health assessment and intervention for at least three health risk or developmental promotion behaviors.		
	**HVE**	Reminders	Uses patient information, clinical data, and evidence-based guidelines to generate lists of patients who need reminders and to proactively remind patients/families/caregivers and clinicians of needed services.	Tracks the number of eligible patients who were sent appropriate reminders.	Sends appropriate reminders to at least 20% of all eligible patients.
**Continuity**	**PCPCH**	Personal Clinician Assigned	N/A	N/A	Meets a benchmark in the percentage of active patients assigned to a personal clinician and/or team.
		Personal Clinician Continuity	N/A	N/A	Meets a benchmark in the percent of patient visits with assigned clinician/team.
		Clinical Information Exchange—shares & tracks electronically	N/A	N/A	Shares clinical information electronically in real time with other providers and care entities (electronic health information exchange).
	**HVE**	Clinical Information Exchange	Exchanges structured clinical information and tracks critical elements (e.g., hospitalizations).		
		Utilization Follow-up	Follows up on patient hospitalizations and ED visits 30% of the time (when they have the information).	Follows up on patient hospitalizations or ED visits 70% of the time (when they have the information).	Follows up on patient hospitalizations and ED visits 70% of the time (when they have the information).

		Utilization Prevention	Selects and reviews utilization measures and goals most relevant to their overall patient panel, or an at-risk patient population.	Shows improvement or meets a benchmark in utilization metrics on measures closely linked to utilization.	
**Coordination & Integration**	**PCPCH**	Population Data Management	Demonstrates the ability to identify, aggregate, and display up-to-date data regarding its patient population.	Demonstrates the ability to identify, track and proactively manage the care needs of a sub-population of its patients using up-to-date information.	
		Clinical Information Exchange—shares electronically	N/A	N/A	Has an electronic health record and demonstrates meaningful use of the electronic record, according to the Centers for Medicare and Medicaid rules.
		Care Coordination—describes process	Assigns individual responsibility for care coordination and tells each patient or family the name of the team member responsible for coordinating his or her care.	Describes and demonstrates its process for identifying and coordinating the care of patients with complex care needs.	

Coordination & Integration	PCPCH	Test & Results Tracking	Demonstrates tracking of tests ordered by its clinicians and ensures timely and confidential notification or availability of results to patients and families with interpretation, as well as to ordering clinicians.		
		Comprehensive Care Planning—demonstrates ability	N/A	Demonstrates the ability to identify patients with high-risk environmental or medical factors, including patients with special health care needs, who will benefit from additional care planning. PCPCH demonstrates it can provide these patients and families with a written care plan that includes the following: self-management goals; goals of preventive and chronic illness care; action plan for exacerbations of chronic illness (when appropriate); end of life care plans (when appropriate).	
		Referral & Specialty Care Coordination	Demonstrates tracking referrals ordered by its clinicians, including referral status and whether consultation results have been communicated to patients and/or caregivers and clinicians.	Either manages hospital or skilled nursing facility care for its patients or demonstrates active involvement and coordination of care when its patients receive care in these specialized care settings.	Tracks referrals and coordinates care where appropriate for community settings outside the PCPCH (such as dental, educational, social service, foster care, public health, or long term care settings).
	HVE	Care Plan Utilization—for a % of high-risk patients	Reports data on care plans provided to high-risk patients.	Provides care plans to > 25% of high-risk patients.	Provides care plans to >50% of high-risk patients.

**Coordination & Integration**	**HVE**	Advance Directive Utilization	Tracks offers of advance directives to patients over 65.	Offers advance directives to at least 30% of patients over 65.	Offers advance directives to at least 50% of patients over 65.
		Performance Data Utilization	Uses performance data to identify opportunities for improvement and acts to improve clinical quality, efficiency and patient experience.		
		Care Coordination Outreach—for a % of high-risk patients	Care coordination outreach reaches 25% of high-risk patients.	Care coordination outreach reaches 50% of high-risk patients.	
**Patient & Family Centered**	**PCPCH**	Education & Self Management Support—documents	Documents patient and family education, health promotion and prevention, and self-management support efforts, including available community resources.		
		Experience of Care	Surveys a sample of its patients and families at least annually on their experience of care. The patient survey must at least include questions on access to care, provider communication, coordination of care, and practice staff helpfulness. The recommended patient experience of care survey is one of the CAHPS survey tools.	Surveys a sample of its population using one of the CAHPS survey tools.	Surveys a sample of its population using one of the CAHPS survey tools and meets benchmarks on a majority of the survey domains.
	**HVE**	Education and Self-management Resources	More than 10% of all unique patients are provided patient-specific education resources.	More than 10% of all unique patients are provided patient-specific education resources and self-management services.	
